# Discovery of novel inhibitors targeting nematode chitinase C*e*Cht1: Virtual screening, biological evaluation, and molecular dynamics simulation

**DOI:** 10.3389/fchem.2022.1021295

**Published:** 2022-11-03

**Authors:** Shengqiang Shen, Baokang Ding, Xi Jiang, Meiling Yang, Qing Yang, Lili Dong

**Affiliations:** ^1^ Academy for Advanced Interdisciplinary Studies, Peking University, Beijing, China; ^2^ State Key Laboratory of North China Crop Improvement and Regulation, College of Plant Protection, Hebei Agricultural University, Baoding, China; ^3^ State Key Laboratory for Biology of Plant Diseases and Insect Pests, Institute of Plant Protection, Chinese Academy of Agricultural Sciences, Beijing, China; ^4^ Guangdong Laboratory for Lingnan Modern Agriculture (Shenzhen Branch), Agricultural Genomics Institute at Shenzhen, Chinese Academy of Agricultural Sciences, Shenzhen, China

**Keywords:** nematicide, chitinase, inhibitor, CeCht1, virtual screening, inhibitory mechanism

## Abstract

Plant-parasitic nematodes are a main limiting factor for worldwide agriculture. To reduce the global burden of nematode infections, chemical nematicides are still the most effective methods to manage nematodes. With the increasing resistance of nematodes, the development of new anti-nematicides drug is urgent. Nematode chitinases are found to play important roles in various physiological functions, such as larva moulting, hatching from eggshell, and host infection. Inhibition of nematode chitinase is considered a promising strategy for the development of eco-friendly nematicides. In this study, to develop novel nematode chitinase *Ce*Cht1 inhibitors, virtual screening of the ZINC database was performed using the pesticide-likeness rules, pharmacophore-based and docking-based approach in turn. Compounds **HAU-4** and **HAU-7** were identified as potent *Ce*Cht1 inhibitors with the IC_50_ values of 4.2 μM and 10.0 μM, respectively. Moreover, molecular dynamics simulations combined with binding free energy and free energy decomposition calculations were conducted to investigate the basis for the potency of the two inhibitors toward *Ce*Cht1. This work gives an insight into the future rational development of novel and potent nematode chitinase inhibitors.

## Introduction

Nematodes occur as parasites in humans, animals and plants or as free-living forms in soil, fresh water, and marine environments (Traunspurger, 2000). Among them, plant-parasitic nematodes (PPNs) represent a significant limiting factor for global agriculture and cause up to $157 billion in economic losses worldwide each year (Abad et al., 2008; Chen J. et al., 2020). The most efficient approach of controlling PPNs is considered to be the use of chemical nematicides (Albonico et al., 1999; Ntalli et al., 2012). However, due to the widespread use of some traditional nematicides, the problem of nematode resistance has become increasingly serious (Abebew et al., 2022; Vanegas et al., 2022). Therefore, the development of green pesticides for novel nematode targets is of great significance for the control of plant-parasitic nematodes.

Chitin, a linear homopolymer of N-acetyl-D-glucosamine, is known to exist in the eggshell, microfilarial sheath, and pharynx of nematodes, but not in higher plants and mammals (Adam et al., 1996; Fanelli et al., 2005; Veronico et al., 2001; Zhang et al., 2005). The chitinase that catalyzes the degradation of nematode chitin is found to play an important role in various physiological functions, including larva moulting, hatching from eggshell, and host infection (Chen W. et al., 2020; Gao et al., 2002; Maeda et al., 2001). For example, using RNAi to silence the expression of *Caenorhabditis elegans* chitinase (*Ce*Cht1) led to hatching failure and eventual nematode death (Maeda et al., 2001). Down-regulating the expression level of *Acanthocheilonema viteae* chitinase (*Av*Cht1) can affect nematode egg hatching and larval molting (Tachu et al., 2008). Downregulation of *Bursaphelenchus xylophilus* chitinase (*Bx*Cht1) led to hatching delay and spawning decrease (Ju et al., 2016). Accordingly, nematode chitinase may serve as a potential target for the development of novel nematicides (Chen W. et al., 2021; Chen Q. et al., 2021).

**FIGURE 1:**
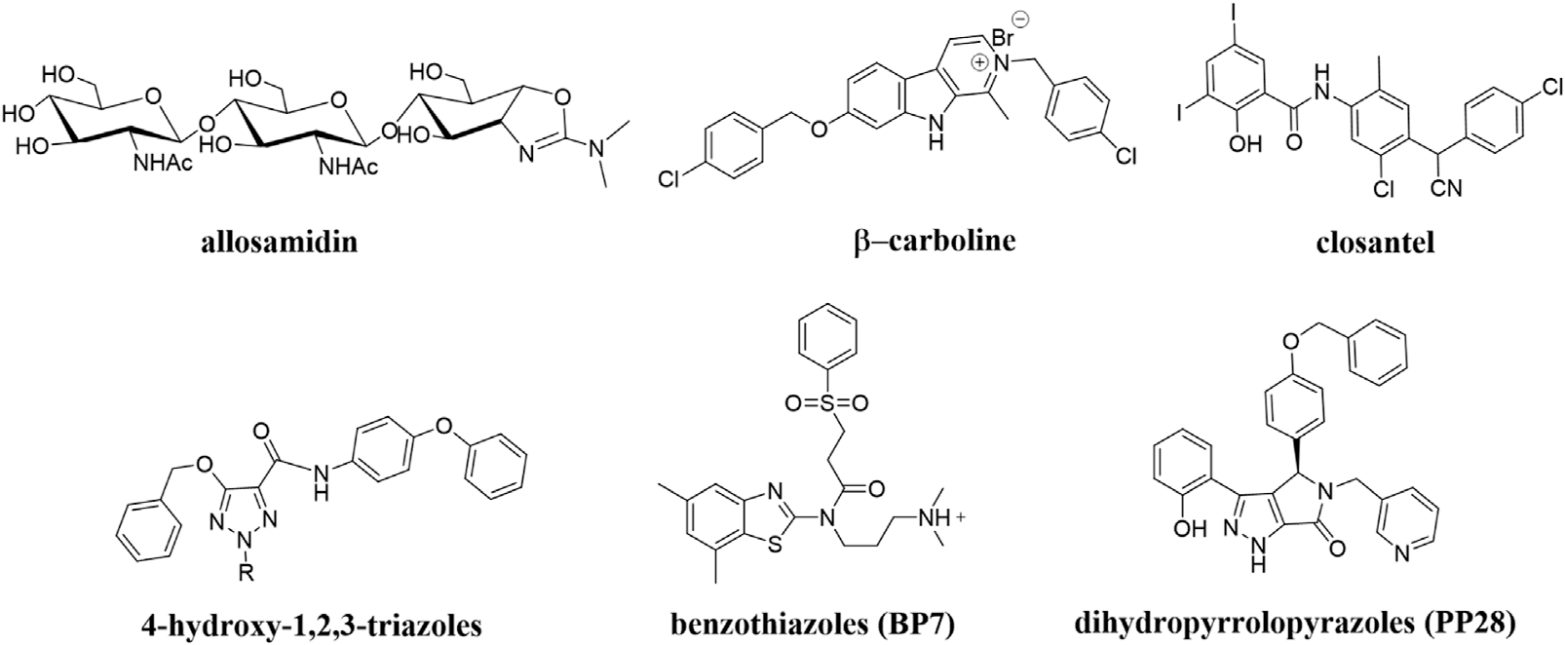
Reported nematode chitinase inhibitors.

To date, a large number of chitinase inhibitors have been reported, and most of which show excellent application prospects in drug, insecticide, and fungicide (Chen J. et al., 2020; Gloeckner et al., 2010; Jiang et al., 2020). However, the development of chitinase inhibitors for nematicides is rarely mentioned. Only few nematode chitinase inhibitors have been reported, including allosamidin (Arnold et al., 1993), *β-*carboline (Gooyit et al., 2015), closantel (Gloeckner et al., 2010), 4-hydroxy-1,2,3-triazoles (Pippione et al., 2015), benzothiazoles (BP) (Chen Q. et al., 2021), and dihydropyrrolopyrazoles (PP) (Chen W. et al., 2021) (Figure 1). Among these inhibitors, allosamidin, carbolines, closantel, and 4-hydroxy-1,2,3-triazoles were reported to have good inhibitory effect on animal parasitic nematodes (e.g., *Heligmosomoides polygyrus*, *Brugia malayi*, *Onchocerca volvulus*). Benzothiazoles (BP) and dihydropyrrolopyrazole (PP) were recently found by Yang’s group to have excellent inhibitory potency against the model nematode *Caenorhabditis elegans*. Furthermore, the crystal structures of *Caenorhabditis elegans* chitinase *Ce*Cht1 and *Ce*Cht1-inhibitor complexes were obtained by Yang’s group (Chen W. et al., 2021; Chen Q. et al., 2021). The results showed that *Ce*Cht1 consists of a signal peptide, a catalytic domain, and two chitin-binding modules. The catalytic signature motif of *Ce*Cht1 is located in the loop between *β*4 and *α*4, and the key catalytic residues is composed of Asp175, Asp177, and Glu179 (Chen Q. et al., 2021). These crystallographic investigations on *Ce*Cht1 lay the solid foundation for the discovery of novel nematode chitinase inhibitors *via* structure-based virtual screening.

*In silico* virtual screening has been rapidly developed as a reliable, timesaving strategy to obtain novel inhibitors that target a given protein of interest (Dong et al., 2019; Gorgulla et al., 2020). In order to acquire more potent nematode chitinase inhibitors with novel chemical scaffolds, especially their application against plant-parasitic nematicides, the virtual screening targeting *Ce*Cht1 was carried out using both pharmacophore-based and docking-based methods in this study. In the subsequent steps, 15 compounds were selected for further enzymatic assay, and a molecule named 1-ethyl-3-((4-methoxyphenethyl) carbamoyl)- 10-methyl- 5-oxo- 1,5-dihydro- 2*H*-dipyrido [1,2-*a*:2′,3′-*d*] pyrimidin-2-iminium was identified as a novel type of *Ce*Cht1 inhibitor. Moreover, to analyze the possible inhibitory mechanisms of potent inhibitors with *Ce*Cht1, MD simulations combined with binding free energy and free energy decomposition calculations were performed. The results obtained in this work provide important insights into the future development of novel and potent plant-parasitic nematodes chitinase inhibitors.

## Results and discussion

### Pharmacophore-based virtual screening

**FIGURE 2:**
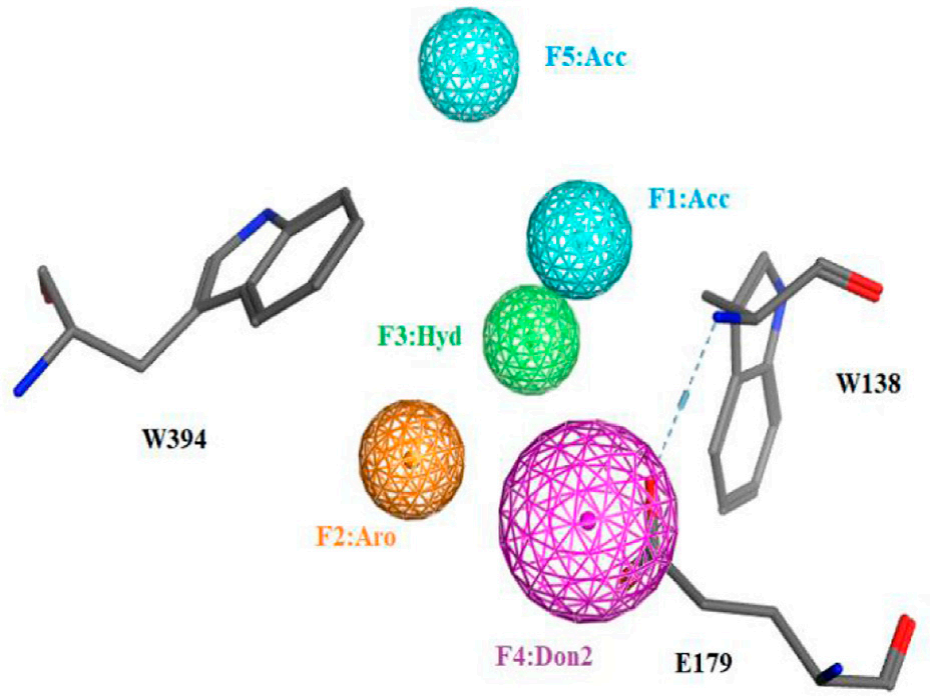
The pharmacophore model used for the virtual screening of *Ce*Cht1 inhibtors. Definitions: Don2, hydrogen-bond donor projection; Acc, hydrogen-bond acceptor; Aro, aromatic center; Hyd, hydrophobic centroid.

To identify novel and potent nematode chitinase inhibitors, in this study, a total of 8 million molecules from ZINC library were used for virtual screening. Firstly, in order to improve the screening efficiency, the pesticide-likeness rules (Hao et al., 2011) were used to reasonably reduce the number of compounds and this yielded 3176361 molecules. Then, the reported co-crystal structure of *Ce*Cht1 with PP7 (PDB ID: 6LE8) (Chen W. et al., 2021) was selected as the template to generate the pharmacophore model in MOE. We focused on the H-bonding and hydrophobic interactions between ligand and residues Glu179, Trp138, and Trp394 at −1 and +1 subsists of *Ce*Cht1. As a result, a pharmacophore model was constructed with the features of a hydrogen bond donor at Glu179, aromatic center at Trp394, a hydrogen-bond acceptor and a hydrophobic center at Trp138 (Figure 2). Based on this pharmacophore model, 1376261 compounds were obtained.

### Docking-based virtual screening

**FIGURE 3:**
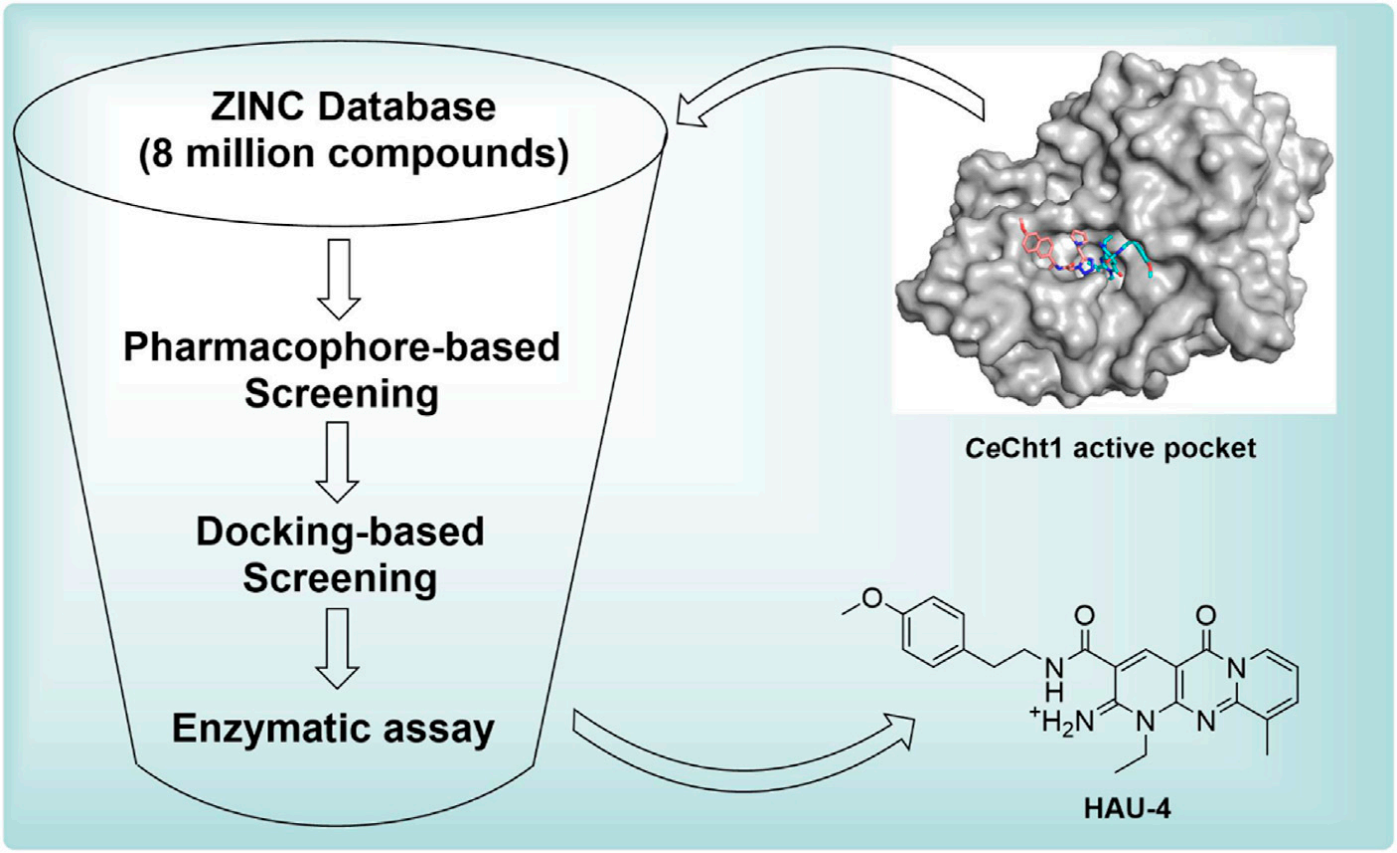
Workflow of the virtual screening.

To further reduce the number of the hits, these molecules were refined with docking-based virtual screening. The virtual screening was performed using the MOE modeling software, and the crystal structure of *Ce*Cht1 complexed with PP7 was selected as the template for molecular docking. The obtained poses were rank-ordered according to their binding scores (a higher negative score indicated a higher stable interaction). As a result, the top 500 compounds with score ranging from −9.75 to −9.00 were selected. These hits were further refined with visual inspection. Considering the interactions between *Ce*Cht1 and the corresponding inhibitors revealed from crystal complexes, only hits that mainly interacted with residues Trp138, Asp177, Asp179, Tyr247, Asp248 and Trp394 were considered. Finally, 15 representative compounds were purchased from Topscience Corporation and were used for biological activity assays. Structures, docking scores of the compounds are listed in Supplementary Table S1. The corresponding sequence of these steps is summarized in Figure 3.

### Enzyme inhibition evaluation

After the 15 potential compounds were purchased, the corresponding inhibitory activities against *Ce*Cht1 were assayed at a concentration of 100 μM. As shown in Supplementary Table S1, compounds **HAU-4**, **HAU-7**, **HAU-9**, and **HAU-11** exhibited good inhibitory potency against *Ce*Cht1 with inhibition rates >75% at 100 μM. Two compounds (namely, **HAU-1** and **HAU-3**) showed moderate efficiency against *Ce*Cht1 with inhibition rates >50% at 100 μM. The remaining compounds showed relatively weak efficiency (inhibition rate <50% at 100 μM). These results preliminarily indicate that our virtual screening strategy is effective.

Seven compounds with inhibition rates >50% at the concentration of 100 μM were further assayed for their inhibitory activities. As shown in Table 1, compounds **HAU-4** (ZINC09610803) and **HAU-7** (ZINC12704597) exhibited excellent potency against *Ce*Cht1 with IC_50_ values of 4.2 μM and 10.0 μM, respectively (Supplementary Figure S1). The remaining compounds displayed inhibition rate <50% at 10 μM against *Ce*Cht1. Therefore, compounds **HAU-4** and **HAU-7** can be used as the novel nematode chitinase inhibitors, and the study of their inhibition mechanisms is of great significance for guiding the development of new nematicides.

**TABLE 1:** Further inhibitory activity assays of representative compounds towards *Ce*Cht1.

Sample NO.	Structure	Inhibition rate at 10 μM (%)	IC_50_ (μM)
**HAU-1**	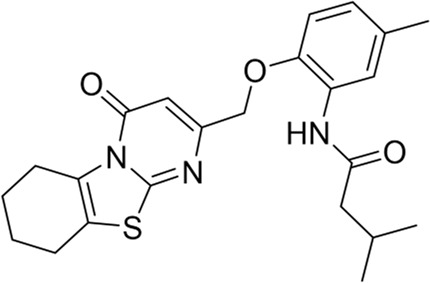	30.4 ± 2.5	Nd
**HAU-3**	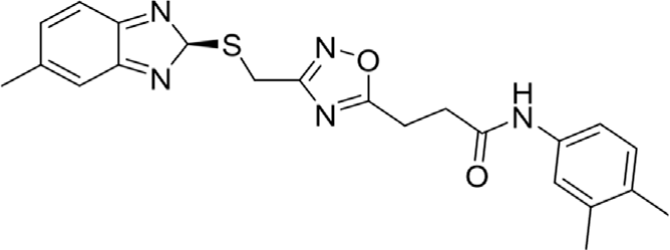	0.4 ± 0.2	Nd
**HAU-4**	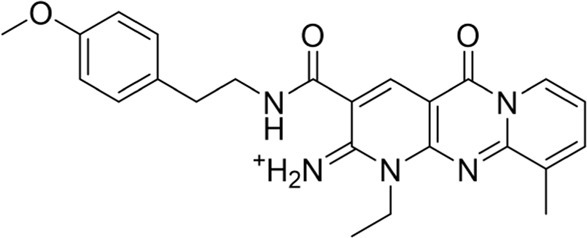	**68.5 ± 1.6**	**4.2 ± 0.5**
**HAU-7**	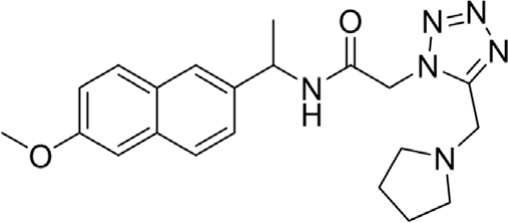	**51.2 ± 4.1**	**10.0 ± 2.6**
**HAU-9**	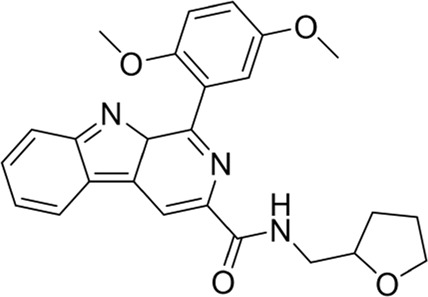	39.4 ± 3.3	Nd
**HAU-11**	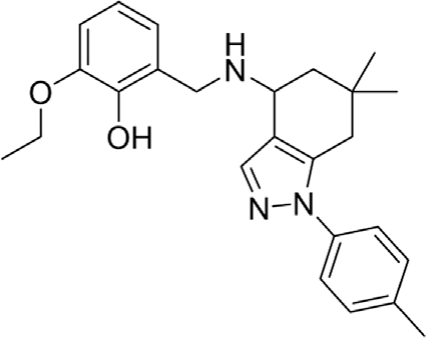	34.9 ± 1.6	Nd
**HAU-15**	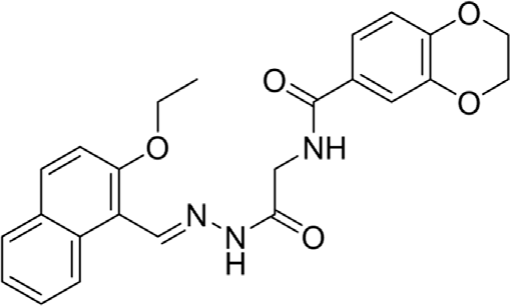	2.0 ± 0.8	Nd

Nd: not determined (less than 50% inhibition at 10 μM); Bold values indicate bioassay data of compounds with better activity.

### Inhibitory mechanisms of HAU-4 and HAU-7 against *Ce*Cht1

To investigate the basis for the potency of compounds **HAU-4** and **HAU-7** toward *Ce*Cht1, the molecular docking studies were carried out. As shown in Supplementary Figure S2A,B,C, compounds **HAU-4** and **HAU-7** were all well-anchored in the substrate-binding pocket of *Ce*Cht1 at −1, +1, and +2 subsites, and stabilized by hydrophobic interactions and hydrogen bonds. Specifically, the dipyrido-pyrimidine moiety of **HAU-4** was found to bind to the -1 and +1 subsites of *Ce*Cht1, and formed H-bonding interactions with residues Trp138 and Asp248 (Supplementary Figure S2B). The *p*-methoxyphenyl fragment of **HAU-4** was located at the +2 subsite of the active pocket entrance consisting of Trp253 (Supplementary Figure S2B). For compound **HAU-7**, the pyrrole ring was shown to be inserted into the -1 subsite of *Ce*Cht1 *via* π-π stacking interactions with Trp394, and the amide linker can form H-bonding interactions with residues Trp138 and Asp248 (Supplementary Figure S2C). In addition, the naphthalene ring of **HAU-7** extends to the +2 subsite (Supplementary Figure S2C).

**FIGURE 4:**
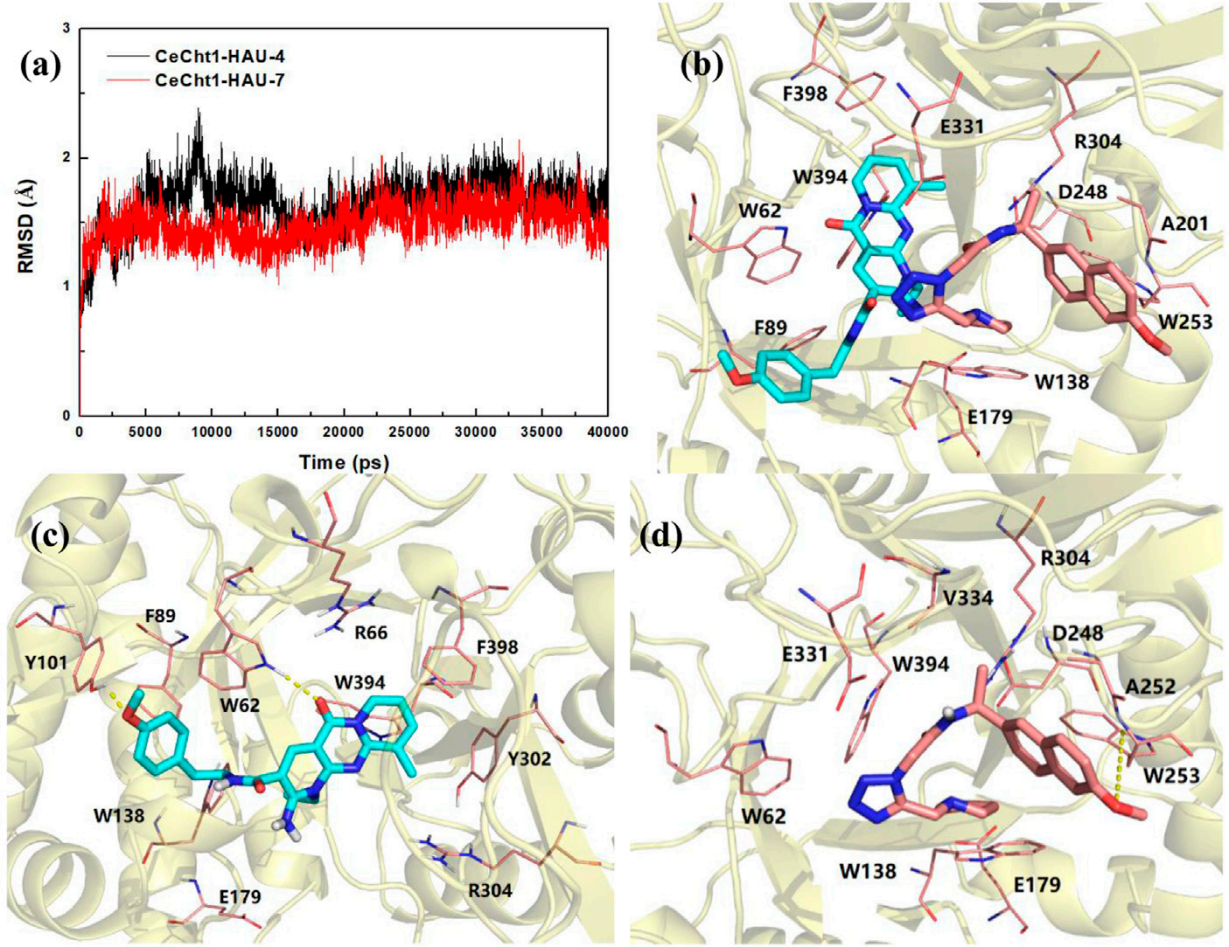
**(A)** RMSD changes of compounds **HAU-4** and **HAU-7** in complex with *Ce*Cht1. **(B)** Superimposition of the conformations of **HAU-4** and **HAU-7** in *Ce*Cht1 pocket at 40 ns MD simulations. Specific binding conformations of **(C)** *Ce*Cht1**-HAU-4** and **(D)** *Ce*Cht1-**HAU-7** systems revealed by MD simulations. Compound **HAU-4** is shown in cyan; **HAU-7** is shown in pink. Colored according to element.

To shed further light on the appropriate binding modes of compounds **HAU-4** and **HAU-7** with *Ce*Cht1 and in an effort to assay the stabilities of the potential compounds in the active pocket, 40 ns MD simulations were performed (Figure 4). As shown in Figure 4A, the two simulated systems all achieved dynamic equilibrium at approximately 25 ns and their root-mean-square deviation (RMSD) values ultimately maintained at 1.7 and 1.5 Å for protein-ligand complexes of compounds **HAU-4** and **HAU-7**, respectively. These results indicated that these two systems underwent reasonable conformational changes.

Superimposition of the conformations of **HAU-4** and **HAU-7** with *Ce*Cht1 at 40 ns of MD simulations are shown in Figure 4B and Supplementary Figure S2D. Compound **HAU-7** was found to be buried at the entrance of the *Ce*Cht1 active pocket, and mainly binds to the +1 and +2 subsites of *Ce*Cht1. In contrast, compound **HAU-4** was found to enter deeper into the active pocket, and the dipyrido-pyrimidine ring was inserted into the -1 subsite of *Ce*Cht1 (Figure 4B and Supplementary Figure S2D).

The specific binding mode of **HAU-4** with *Ce*Cht1 revealed by MD simulations is shown in Figure 4C. In comparison to the docking conformation (Supplementary Figure S2B), the dipyrido-pyrimidine ring of **HAU-4** was demonstrated to move deeper into the *Ce*Cht1 pocket *via* 40 ns MD simulations, and the *p*-methoxyphenyl moiety was far from the +1 and +2 subsites (Supplementary Figure S2E). In detail, the dipyrido-pyrimidine moiety of **HAU-4** stacked well with Trp394, Phe398, Tyr302, and Trp62 at −1 subsite. The oxygen atom on dipyrido-pyrimidine ring formed a hydrogen bond with Trp62. On the other hand, the *p*-methoxyphenyl fragment of **HAU-4** bound in a small hydrophobic cave constructed by residues Tyr101, Phe89, Trp138, and it was further stabilized by forming a hydrogen bond with Tyr101. Furthermore, we found that the ethyl group on the dipyrido-pyrimidine points to the position of the catalytic residues Asp175, Asp177, and Glu179 (Chen Q. et al., 2021). This may suggest the introduction of suitable substituent (such as fragment with hydrogen bond donor) in the ethyl of **HAU-4** may lead to increased activity. Thus, this binding pattern provided a clear molecular basis for the potent inhibitory activity of compound **HAU-4** (IC_50_ = 4.2 μM) against *Ce*Cht1.

The conformations of compound **HAU-7** before and after MD simulations in *Ce*Cht1 pocket are superimposed and shown in Supplementary Figure S2F. Compound **HAU-7** was found to exhibit a great deal of folding and move to the entrance of the pocket relative to the conformation before MD simulations (Figure 4D, Supplementary Figure S2C,F). The specific binding mode of **HAU-7** with *Ce*Cht1 revealed by MD simulations showed that the pyrrole moiety anchored in the hydrophobic pocket and formed π-π stacking interactions with Trp138, while the naphthalene ring interacted with Ala252 and Trp253 at +2 subsite of *Ce*Cht1. It is worth noting that **HAU-7** could only form one hydrogen bond with Trp253, with reduced interactions in its docking mode (Figure 4D and Supplementary Figure S2C). Therefore, the affinity between **HAU-7** with *Ce*Cht1 was determined to be relatively weak, resulting in a moderate inhibitory potency against *Ce*Cht1 (IC_50_ = 10.0 μM).

### Binding free energy calculation

To further explore the cause of the inhibitory potency against *Ce*Cht1, binding free energy analyses of **HAU-4** and **HAU-7** were conducted by using MM/GBSA calculation methods. The predicted binding affinities of these complexes are summarized in Table 2. The results showed that the binding free energy (Δ*G*_TOT_) were in agreement with the experimental values of **HAU-4** and **HAU-7**. Furthermore, both of the electrostatic (*E*_ELE_) and the van der Waals (*E*_VDW_) contributions are essential for the two compounds when bound to *Ce*Cht1. For compound **HAU-4**, the electrostatic energy (*E*_ELE_ = −30.53 kcal/mol) was lower than its van der Waals energy (*E*_VDM_ = −29.42 kcal/mol). On the contrary, the van der Waals interactions (*E*_VDM_ = −26.63) of **HAU-7** acted a more important role than the electrostatic interaction kcal/mol (*E*_ELE_ = −13.83 kcal/mol). Moreover, the nonpolar interactions (*E*_VDM_ + *G*_SA_) of these two complexes were lower than the polar interactions (*E*_ELE_ + *G*_GB_), which suggested that the nonpolar interactions in these two systems were more beneficial for the binding of **HAU-4** and **HAU-7** to *Ce*Cht1.

**TABLE 2:** Binding free energy of **HAU-4** and **HAU-7** calculated by MM/GBSA (kcal/mol).

Compd	IC_50_ (μM)	*E*_VDW_	*E*_ELE_	*G*_SA_	*G*_GB_	Δ*G*_TOT_	STD
**HAU-4**	4.2	−29.42	−30.53	−5.25	43.44	−21.76	3.42
**HAU-7**	10.0	−26.63	−13.85	−4.86	28.73	−16.62	3.14

### Decomposition free energy calculation

**FIGURE 5:**
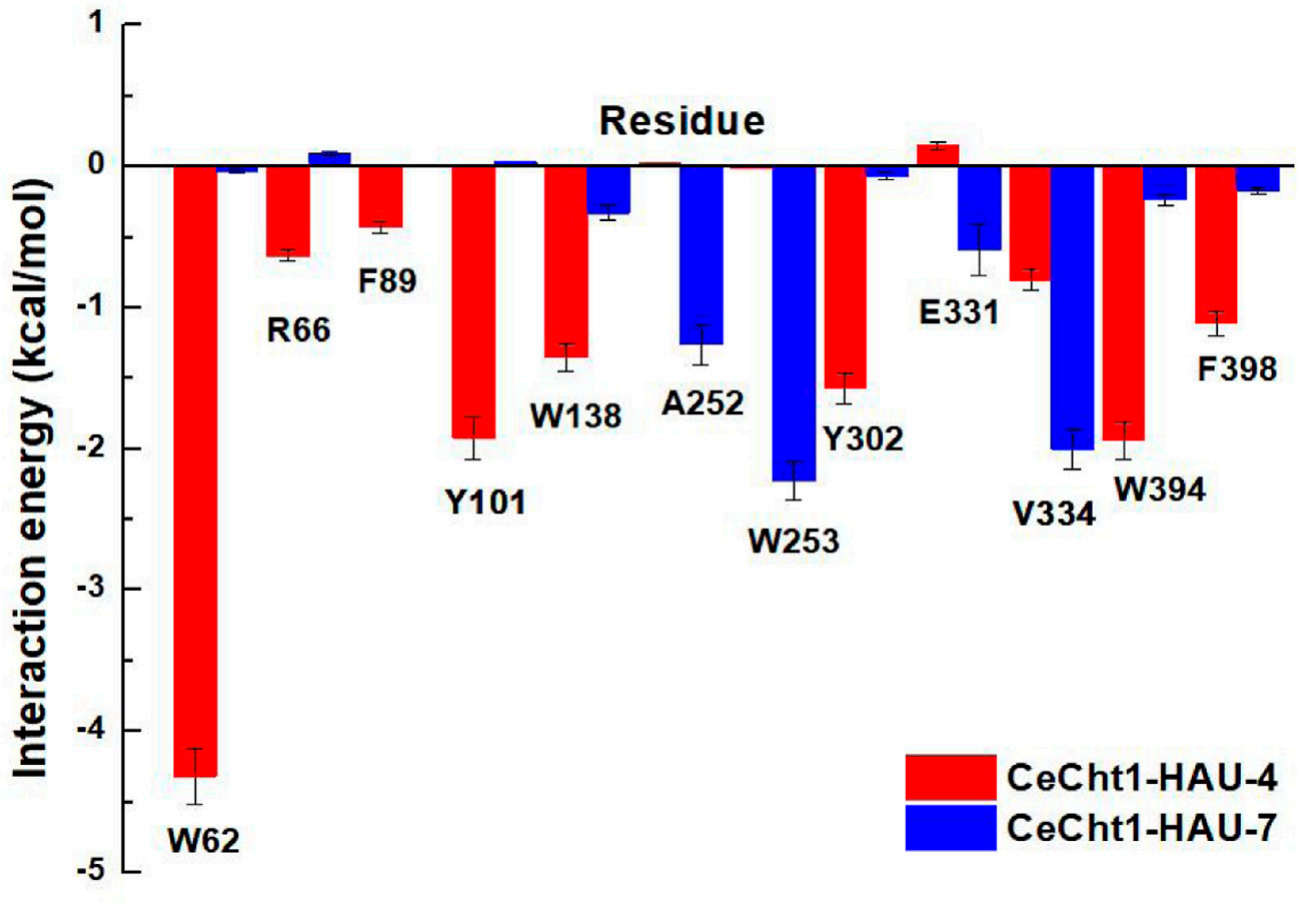
Decomposition free energy of *Ce*Cht1 with **HAU-4** and **HAU-7** calculated by MM-GBSA.

Free energy decomposition calculations were further performed by using MM/GBSA methods to investigate the detailed contributions of residues in the two systems. Residues near 5 Å of the ligands were selected to compute the decomposition free energy, and the main residue contributions are shown in Figure 5. Compound **HAU-4** formed the strongest interaction with Trp62 (−4.32 kcal/mol) and formed moderate interactions with Trp394, Tyr101, Tyr302, and Trp138 **(**−1.94, −1.92, −1.57, and −1.35 kcal mol^−1^, respectively). This suggested that the hydrophobic interactions and hydrogen-bonding interactions at −1 subsite were the main reasons for the higher inhibitory potency of compound **HAU-4**. Compound **HAU-7** mainly interacted with Trp253, Val334, and Ala252 at the entrance of the active pocket. This result further demonstrated the shallow binding mode of **HAU-7** in the *Ce*Cht1 active pocket. It is noteworthy that neither compound bound to the catalytic residue Asp175, Asp177, and Glu179 (Chen Q. et al., 2021), illustrating that the efficiency of compounds **HAU-4** and **HAU-7** is in the micromolar range.

## Conclusion

In conclusion, in order to develop novel nematicides with new mechanisms of action, in this study, we conducted pharmacophore-based and docking-based virtual screening methodology to identify novel and potent inhibitors targeting the model nematode chitinase *Ce*Cht1. As a result, 15 representative compounds from the ZINC database were selected for enzymatic evaluation, and **HAU-4** and **HAU-7** demonstrated the higher potency against *Ce*Cht1 with the IC_50_ values of 4.2 μM and 10.0 μM, respectively. Furthermore, MD simulations combined with binding free energy and free energy decomposition calculations of these two inhibitors with *Ce*Cht1 were carried out to investigate the basis for their potency against *Ce*Cht1. These computational study results also suggest that introducing a suitable substituent (such as fragment with hydrogen bond donor) on the ethyl group of dipyrido-pyrimidine ring of **HAU-4** may result in additional bindings with catalytic residues of *Ce*Cht1 and ultimately improve the inhibitory activity. Taken in concert, the combined theoretical and experimental results in this manuscript may provide a new direction for the future discovery of potent nematodes chitinase inhibitors.

## Materials and methods

### Preparation of the Zinc database

The ZINC database containing 8 million molecules was used for our virtual screening targeting nematode chitinase *Ce*Cht1. Initially, the descriptors of all the molecules in the ZINC library were calculated *via* the Descriptor module in MOE software (version 2016). Secondly, these molecules were filtered based on “Hao’s pesticide-likeness” rules (Hao et al., 2011), which are MW < 435 Da, ClogP <6, HBA <6, HBD <2, ROB <9, ARB <17. In addition, the synthetic feasibility score in MOE of these compounds was considered. The synthetic feasibility score is the fraction of atoms of each new structure that ultimately appear in a retrosynthetic fragment, and a value of one means the molecule is likely to be synthesizable. Based on these criteria, the unsuitable molecules were gradually filtered out.

### Pharmacophore-based screening

The complex crystal structure of *Ce*Cht1 (PDB ID: 6LE8) (Chen W. et al., 2021) was selected as the template to generate a pharmacophore model. The protein ligand interaction fingerprints (PLIF) tool in MOE was used to establish a pharmacophore model. The pharmacophore features take the spatial position and interactions between ligands and proteins into account, including hydrogen bond donor and acceptor, and hydrophobic interaction characteristics. Then, virtual screening module in MOE was used to perform the pharmacophore-based screening.

### Docking-based screening

Molecular docking was carried out by using the Dock module in MOE (Dong et al., 2021). The complex crystal structure of *Ce*Cht1 with PP7 (PDB ID: 6LE8) (Chen W. et al., 2021) was used as the template for the docking-based screening, and the position of the ligand was defined as the docking site. First, the Structure Preparation module in MOE was used to prepare the protein structure. The typical steps in this process included the geometry and electron-density checks, addition of hydrogen atoms, optimization of their position, and energy minimization. Then, the Triangle Matcher placement method and Rigid Receptor post-placement refinement method were used to generate the poses of ligand. The number of poses returned by each ligand placement were set to a default value of 30. The London dG scoring function was used to estimate their free energy of binding. The other parameters were set default values. The molecules were ranked lowest to highest according to their scores, and the pose with the lowest score was retained.

### Enzymatic assays

Nematode chitinase *Ce*Cht1 (*Ce*Cht1-CAD) was overexpressed in *Pichia pastoris GS115* (Invitrogen, Carlsbad, CA) and then purified as described previously (Chen Q. et al., 2021).

The target compounds were assayed for their inhibitory activities against *Ce*Cht1 in end-point experiments using 4-methylumbelliferyl -N, N′-diacetyl-β-D-chitobioside (4-MU-(GlcNAc)_2_, Sigma, St. Louis, MO) as the substrate. The *Ce*Cht1 was assayed in 20 mM sodium phosphate buffer (pH 6.0). In a final volume of 100 μl, the reaction mixture containing buffer, DMSO (1% (v/v)), *Ce*Cht1-CAD (10 nM), 4-MU- (GlcNAc)_2_ (4 μM) and inhibitor was incubated at 25°C for 20 min. Then, the reaction was stopped by the addition of sodium carbonate solution (0.5 M, 100 μl). The fluorescence of the liberated 4-methylumbelliferone was quantitated at an excitation of 366 nm and emission of 440 nm. Experiments were performed in triplicate. For determination of the IC_50_ values, the inhibitory activity of the compounds against *Ce*Cht1 was monitored by changing the concentrations of inhibitors.

### Molecular dynamics simulations

Molecular Dynamics (MD) simulations were carried out to assess the validity of the binding interactions of the selected compounds and *Ce*Cht1 by using AMBER14 package. The ligands and protein were selected for GAFF force field (Duan et al., 2003) and AMBER03 force field (Wang et al., 2004), respectively. The protein-inhibitor system was immersed in a radius truncated octahedral box with TIP3P water molecules extended 10 Å from the complex, and the system was neutralized by adding counterions (Na^+^). Then, two-step energy minimization was performed using the first 2,500 steps steepest-descent and last 2,500 steps conjugated gradient algorithms in Sander module. Next, the system was gradually heated from 0 to 300 K in the NVT ensemble and the temperature was maintained at 300 K during 100 ps with a restrain force constant of 5 kcal/mol/Å^2^. The SHAKE algorithm was used to constrain the hydrogen atoms (Ryckaert et al., 1977), and the particle mesh Ewald algorithm was applied to calculate the long-range electrostatic interactions with default cutoff of 8.0 Å under periodic boundary conditions (Darden et al., 1993). Finally, 40 ns MD simulations were carried out at the constant temperature and pressure (300 K and 105 Pa) using the PMEMD module in the AMBER14.

### MM/GBSA calculations

The molecular mechanics/generalized Born surface area (MM-GBSA) molecular method was performed to calculate the binding free energy (Δ*G*_bind_) in the post-processing trajectory analysis in Amber14. The last 5 ns simulations were selected as the binding equilibrium conformation to calculate binding free energy. The Δ*G*_bind_ of *Ce*Cht1-inhibitor complexes was evaluated by energy minimization of the ligand, receptor, and the complex structure as follows:

$$\begin{array}{l} ∆G_{bind}=G_{complex}–\left(G_{receptor}+G_{ligand}\right),\left(1\right)\\ ∆G_{bind}=∆E_{MM}+∆G_{solvation}–T∆S,\left(2\right)\\ ∆E_{MM}=∆E_{internal}+∆E_{electrostatic}+∆E_{VDW},\left(3\right)\\ ∆G_{solvation}=∆G_{polar\;sol}+∆G_{\text{nonpolarsol}},\left(4\right) \end{array}$$
where *G*_complex_, G_receptor_, and G_ligand_ represent the free energy of complex, *Ce*Cht1, and ligand, respectively. Δ*G*_bind_ was evaluated by gasphase interaction energy (Δ*E*_MM_), solvation energy term (Δ*G*_solvation_) and entropy term (TΔ*S*) between the candidate molecules and *Ce*Cht1. ΔE_MM_ is the gasphase interaction energy and it contains internal (Δ*E*_internal_), electrostatic (Δ*E*_electrostatic_), and van der Waals energies (Δ*E*_VDW_). Δ*G*_solvation_ is the solvation free energy, which is the sum of the polar solvation contribution and nonpolar solvation contributions. TΔ*S* shows the change of conformational entropy upon ligand binding. The energy decomposition was carried out to evaluate the contribution of each residue of the *Ce*Cht1 *via* the MM-GBSA method in AMBER14.

## Data Availability

The original contributions presented in the study are included in the article/Supplementary Material, further inquiries can be directed to the corresponding authors.

## References

[B1] Abad P. Gouzy J. Aury J.-M. Castagnone-Sereno P. Danchin E. G. J. Deleury E. (2008). Genome sequence of the metazoan plant-parasitic nematode Meloidogyne incognita. Nat. Biotechnol. 26, 909–915. 10.1038/nbt.1482 18660804

[B2] Abebew D. Sayedain F. S. Bode E. Bode H. B. (2022). Uncovering nematicidal natural products from xenorhabdus bacteria. J. Agric. Food Chem. 70, 498–506. 10.1021/acs.jafc.1c05454 34981939 PMC8778618

[B3] Adam R. Kaltmann B. Rudin W. Friedrich T. Marti T. Lucius R. (1996). Identification of chitinase as the immunodominant filarial antigen recognized by sera of vaccinated rodents. J. Biol. Chem. 271 (3), 1441–1447. 10.1074/jbc.271.3.1441 8576136

[B4] Albonico M. Crompton D. W. Savioli L. (1999). Control strategies for human intestinal nematode infections. Adv. Parasitol. 42, 277–341. 10.1016/s0065-308x(08)60151-7 10050275

[B5] Arnold K. Brydon L. J. Chappell L. H. Gooday G. W. (1993). Chitinolytic activities in Heligmosomoides polygyrusand their role in egg hatching. Mol. Biochem. Parasitol. 58 (2), 317–323. 10.1016/0166-6851(93)90054-2 8479456

[B6] Chen J. Li Q. X. Song B. (2020). Chemical nematicides: Recent research progress and outlook. J. Agric. Food Chem. 68, 12175–12188. 10.1021/acs.jafc.0c02871 33079521

[B7] Chen W. Jiang X. Yang Q. (2020). Glycoside hydrolase family 18 chitinases: The known and the unknown. Biotechnol. Adv. 43, 107553. 10.1016/j.biotechadv.2020.107553 32439576

[B8] Chen W. Chen Q. Kumar A. Jiang X. Zhang K. Y. J. Yang Q. (2021). Structure-based virtual screening of highly potent inhibitors of the nematode chitinase CeCht1. J. Enzyme Inhib. Med. Chem. 36, 1198–1204. 10.1080/14756366.2021.1931862 34074203 PMC8174485

[B9] Chen Q. Chen W. Kumar A. Jiang X. Janezic M. Zhang K. Y. J. (2021). Crystal structure and structure-based discovery of inhibitors of the nematode chitinase CeCht1. J. Agric. Food Chem. 69 (11), 3519–3526. 10.1021/acs.jafc.1c00162 33691404

[B10] Darden T. York D. Pedersen L. (1993). Particle mesh Ewald: An N·log(N) method for Ewald sums in large systems. J. Chem. Phys. 98 (12), 10089–10092. 10.1063/1.464397

[B11] Dong L. Shen S. Chen W. Xu D. Yang Q. Lu H. (2019). Discovery of novel inhibitors targeting human O-GlcNAcase: Docking-based virtual screening, biological evaluation, structural modification, and molecular dynamics simulation. J. Chem. Inf. Model 59, 4374–4382. 10.1021/acs.jcim.9b00479 31487462

[B12] Dong L. Shen S. Xu Y. Wang L. Yang Q. Zhang J. (2021). Identification of novel insect β-N-acetylhexosaminidase OfHex1 inhibitors based on virtual screening, biological evaluation, and molecular dynamics simulation. J. Biomol. Struct. Dyn. 39, 1735–1743. 10.1080/07391102.2020.1743758 32193983

[B13] Duan Y. Wu C. Chowdhury S. Lee M. C. Xiong G. Zhang W. (2003). A point-charge force field for molecular mechanics simulations of proteins based on condensed-phase quantum mechanical calculations. J. Comput. Chem. 24, 1999–2012. 10.1002/jcc.10349 14531054

[B14] Fanelli E. Di Vito M. Jones J. T. De Giorgi C. (2005). Analysis of chitin synthase function in a plant parasitic nematode, Meloidogyne artiellia, using RNAi. Gene 349, 87–95. 10.1016/j.gene.2004.11.045 15777697

[B15] Gao B. Allen R. Maier T. McDermott J. P. Davis E. L. Baum T. J. (2002). Characterisation and developmental expression of a chitinase gene in Heterodera glycines. Int. J. Parasitol. 32, 1293–1300. 10.1016/S0020-7519(02)00110-8 12204229

[B16] Gloeckner C. Garner A. L. Mersha F. Oksov Y. Tricoche N. Eubanks L. M. (2010). Repositioning of an existing drug for the neglected tropical disease Onchocerciasis. Proc. Natl. Acad. Sci. U. S. A. 107 (8), 3424–3429. 10.1073/pnas.0915125107 20142509 PMC2840490

[B17] Gooyit M. Tricoche N. Javor S. Lustigman S. Janda K. D. (2015). Exploiting the polypharmacology of ß-carbolines to disrupt O. Volvulus molting. ACS Med. Chem. Lett. 6 (3), 339–343. 10.1021/ml500516r 25815157 PMC4360149

[B18] Gorgulla C. Boeszoermenyi A. Wang Z.-F. Fischer P. D. Coote P. W. Padmanabha Das K. M. (2020). An open-source drug discovery platform enables ultra-large virtual screens. Nature 580, 663–668. 10.1038/s41586-020-2117-z 32152607 PMC8352709

[B19] Hao G. Dong Q. Yang G. (2011). A comparative study on the constitutive properties of marketed pesticides. Mol. Inf. 30, 614–622. 10.1002/minf.201100020 27467161

[B20] Jiang X. Kumar A. Motomura Y. Liu T. Zhou Y. Moro K. (2020). A series of compounds bearing a dipyrido-pyrimidine scaffold acting as novel human and insect pest chitinase inhibitors. J. Med. Chem. 63, 987–1001. 10.1021/acs.jmedchem.9b01154 31928006

[B21] Ju Y. Wang X. Guan T. Peng D. Li H. (2016). Versatile glycoside hydrolase family 18 chitinases for fungi ingestion and reproduction in the pinewood nematode Bursaphelenchus xylophilus. Int. J. Parasitol. 46, 819–828. 10.1016/j.ijpara.2016.08.001 27641827

[B22] Maeda I. Kohara Y. Yamamoto M. Sugimoto A. (2001). Large-scale analysis of gene function in Caenorhabditis elegans by high-throughput RNAi. Curr. Biol. 11, 171–176. 10.1016/s0960-9822(01)00052-5 11231151

[B23] Ntalli N. G. Caboni P. (2012). Botanical nematicides: A review. J. Agric. Food Chem. 60, 9929–9940. 10.1021/jf303107j 22973877

[B24] Pippione A. C. Dosio F. Ducime A. Federico A. Martina K. Sainas S. (2015). Substituted 4-hydroxy-1, 2, 3-triazoles: Synthesis, characterization and first drug design applications through bioisosteric modulation and scaffold hopping approaches. MedChemComm 6 (7), 1285–1292. 10.1039/C5MD00182J

[B25] Ryckaert J. P. Ciccotti G. Berendsen H. J. C. (1977). Numerical integration of the cartesian equations of motion of a system with constraints: Molecular dynamics of n-alkanes. J. Comput. Phys. 23, 327–341. 10.1016/0021-9991(77)90098-5

[B26] Tachu B. Pillai S. Lucius R. Pogonka T. (2008). Essential role of chitinase in the development of the filarial nematode Acanthocheilonema viteae. Infect. Immun. 76, 221–228. 10.1128/iai.00701-07 17938220 PMC2223640

[B27] Traunspurger W. (2000). The biology and ecology of lotic nematodes. Freshw. Biol. 44, 29–45. 10.1046/j.1365-2427.2000.00585.x

[B28] Vanegas J. A. G. Pacule H. B. Capitao R. M. Correia C. R. D. Terra W. C. Campos V. P. (2022). Methyl esters of (E)-Cinnamic acid: Activity against the plant-parasitic nematode Meloidogyne incognita and in silico interaction with histone deacetylase. J. Agric. Food Chem. 70 (22), 6624–6633. 10.1021/acs.jafc.1c08142 35622462

[B29] Veronico P. Gray L. J. Jones J. T. Bazzicalupo P. Arbucci S. Cortese M. R. (2001). Nematode chitin synthases: Gene structure, expression and function in Caenorhabditis elegans and the plant parasitic nematode Meloidogyne artiellia. Mol. Genet. Genomics 266, 28–34. 10.1007/s004380100513 11589574

[B30] Wang J. Wolf R. M. Caldwell J. W. Kollman P. A. Case D. A. (2004). development and testing of a general amber force fieldJournal of computational chemistry. J. Comput. Chem. 26, 1157–1174. 10.1002/jcc.20145 15116359

[B31] Zhang Y. Foster J. M. Nelson L. S. Ma D. Carlow C. K. S. (2005). The chitin synthase genes chs-1 and chs-2 are essential for C. elegans development and responsible for chitin deposition in the eggshell and pharynx, respectively. Dev. Biol. 285, 330–339. 10.1016/j.ydbio.2005.06.037 16098962

