# Supplementary material for: Discovery of novel inhibitors targeting nematode chitinase CeCht1: Virtual screening, biological evaluation, and molecular dynamics simulation
Source: Front Chem. 2022 Nov 3;10:1021295. doi: 10.3389/fchem.2022.1021295 (PMC9669442; doi:10.3389/fchem.2022.1021295)
Supplement: Supplementary file 1 [file DataSheet1.PDF]

Supporting information

Discovery of Novel Inhibitors Targeting Nematode Chitinase CeCht1: Virtual Screening, Biological Evaluation, and Molecular Dynamics Simulation

Shengqiang Shen,[a] Xi Jiang,[c,d,] Qing Yang,[*,c,d] Lili Dong,[*,b]

[a] Academy for Advanced Interdisciplinary Studies, Peking University, Beijing 100091, China

[b] State Key Laboratory of North China Crop Improvement and Regulation, College of Plant Protection, Hebei Agricultural University, Baoding 071001, China

[c] State Key Laboratory for Biology of Plant Diseases and Insect Pests, Institute of Plant Protection, Chinese Academy of Agricultural Sciences, Beijing 100193, China

[d] Guangdong Laboratory for Lingnan Modern Agriculture (Shenzhen Branch), Agricultural Genomics Institute at Shenzhen, Chinese Academy of Agricultural Sciences, Shenzhen 518120, China

Content

1. Structures, docking scores and inhibition rate of the selected compounds. 3
2. IC_{50} values of compounds HAU-4 and HAU-7 against *Ce*Cht1. 5
3.The binding mode of HAU-4 and HAU-7 with *Ce*Cht1 revealed by molecular docking and MD simulations 6

1. Structures, docking scores and inhibition rate of the selected compounds.

Table S1. Structures, docking scores and inhibition rate of the compounds against *Ce*Cht1

**Sample NO.**	**ZINC code**	**Structure**	**Docking score**	**Inhibition rate at 100 µM (%)**
**HAU-1**	ZINC35447978	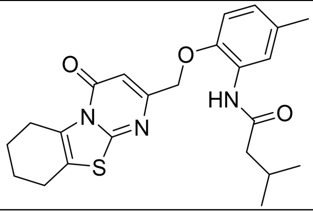	-9.75	**69.3**
**HAU-2**	ZINC63743200	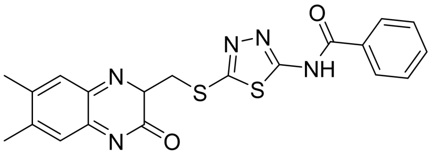	-9.71	46.7
**HAU-3**	ZINC64604304	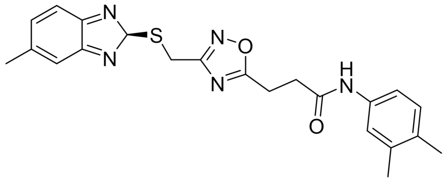	-9.63	**64.2**
**HAU-4**	ZINC09610803	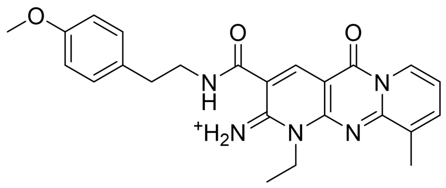	-9.60	**82.3**
**HAU-5**	ZINC03007991	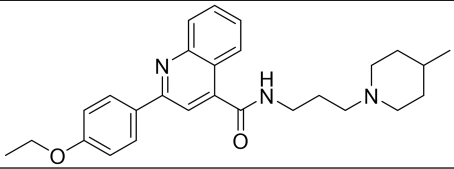	-9.51	2.0
**HAU-6**	ZINC16652377	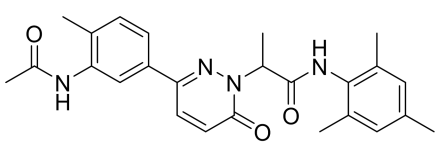	-9.50	0
**HAU-7**	ZINC12704597	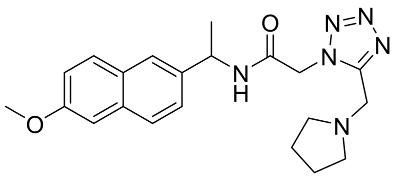	-9.48	**76.3**

**HAU-8**	ZINC63076977	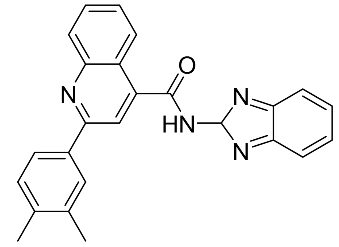	-9.42	38.7
**HAU-9**	ZINC38564275	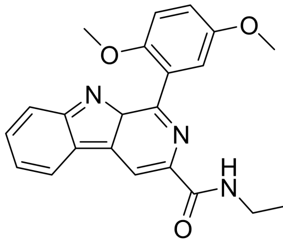	-9.30	**86.2**
**HAU-10**	ZINC12208604	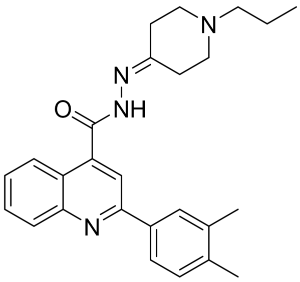	-9.25	0
**HAU-11**	ZINC12208604	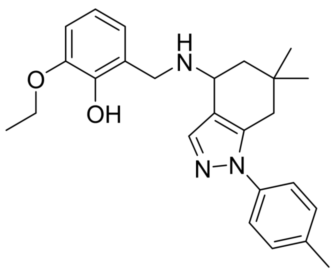	-9.22	**87.4**
**HAU-12**	ZINC12932719	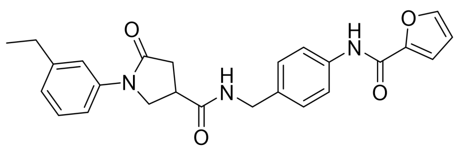	-9.14	0
**HAU-13**	ZINC09575976	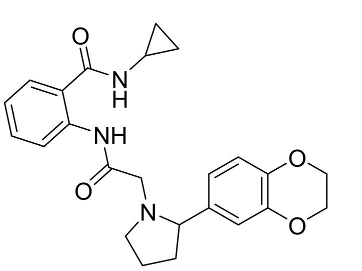	9.06	0
**HAU-14**	ZINC06623856	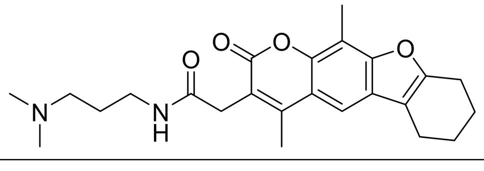	9.05	0

**HAU-15**	ZINC02268638	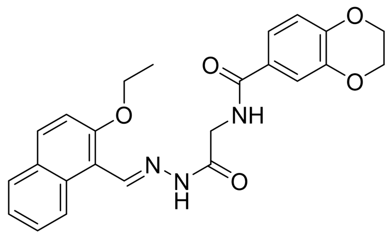	9.01	**88.0**

2. IC_{50} values of compounds HAU-4 and HAU-7 against *Ce*Cht1.

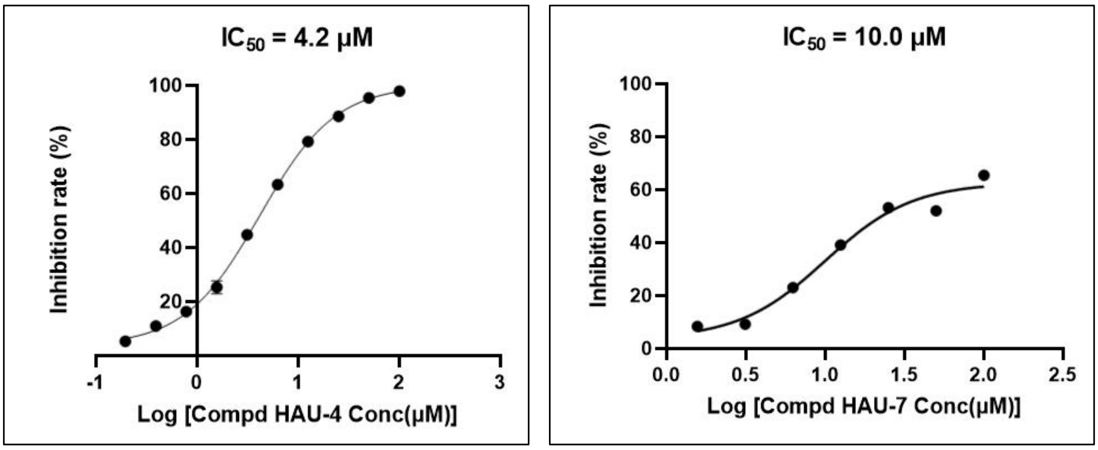

Figure S1. IC_{50} values of compounds **HAU-4** and **HAU-7** against *Ce*Cht1.

3.The binding mode of HAU-4 and HAU-7 with *Ce*Cht1 revealed by molecular docking and MD simulations

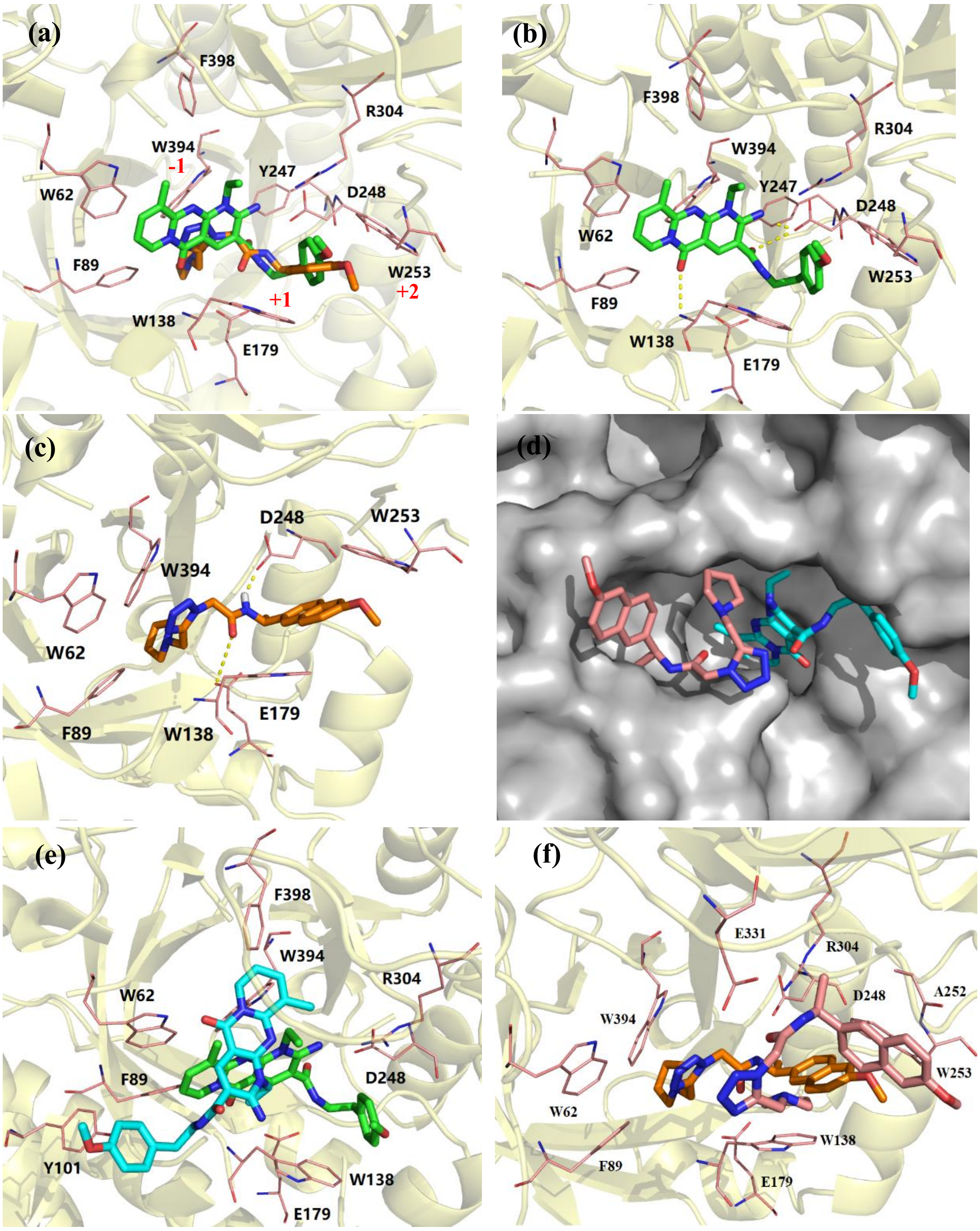

Figure S2. (**a**) Superimposition of conformations of **HAU-4** (colored in green) and **HAU-7** (colored in orange) in *Ce*Cht1 pocket revealed by molecular docking. (**b**) Specific binding modes of **HAU-4** with *Ce*Cht1 revealed by molecular docking. (**c**) Specific binding modes of **HAU-7** with *Ce*Cht1 revealed by molecular docking. (**d**) Superimposition of conformations of **HAU-4** and **HAU-7** in *Ce*Cht1 pocket at 40 ns MD simulations (the enzyme *Ce*Cht1 is presented in surface form). (**e**) Superimposition of conformations of **HAU-4** with *Ce*Cht1 revealed by molecular docking (colored in green) and MD simulations (colored in cyan). (**f**) Superimposition of conformations of **HAU-7** with *Ce*Cht1 revealed by molecular docking (colored in orange) and MD simulations (colored in pink).
